# Application of proteomics to understand the molecular mechanisms determining meat quality of beef muscles during postmortem aging

**DOI:** 10.1371/journal.pone.0246955

**Published:** 2021-03-01

**Authors:** Bin Yang, Xuejun Liu

**Affiliations:** 1 Changchun University, Changchun City, Jilin Province, China; 2 Jilin Agricultural University, Changchun City, Jilin Province, China; University of Illinois, UNITED STATES

## Abstract

Proteomics profiling disclosed the molecular mechanism underlying beef poor meat quality. This study aimed to identify protein markers indicating the quality of beef during postmortem storage at 4°C. Beef longissimus dorsi samples were stored at 4°C. The meat water holding capacity (WHC), pH value and moisture content were determined at different time points during the storage period. The iTRAQ MS/MS approach was used to determine the proteomics profiling at 0, 3.5 and 7 d during storage at 4°C. Bioinformatics analysis was performed to investigate the potential correlated proteins associated with meat quality. Storage at 4°C gradually decreased the pH value, WHC, and hence the moisture content. The iTRAQ proteomic analysis revealed that a cluster of glycolytic enzymes including malate dehydrogenase, cytoplasmic, L-lactate dehydrogenase, phosphoglycerate mutase and pyruvate kinase, and another cluster of proteins involved in oxygen transport and binding (myoglobin) and hemoglobin complex (including Globin A1 and hemoglobin subunit alpha) were decreased during the postmortem storage. These results suggest that the decreased glycolysis, oxygen, and heme-binding activities might be associated with the beef muscle low quality and the decline of tenderness during postmortem storage at 4°C.

## Introduction

The freshness of meat is a major factor influencing the purchasing decisions [1]. Many extrinsic factors including storage methods and temperature and intrinsic factors influence the quality of meat [2–6]. Appearance, texture, tenderness, and flavor are the most important and perceptible features that influence the quality and consumer judgment of a meat product [7].

Proteomics analysis of factors associated with meat quality has implied that storage methods or intrinsic factors influence the meat quality and color stability [8–10]. Proteomics elucidates the organism systems underlying the characterization of foods from a molecular and biological point of view. It has been reported by proteomics that the proteins associated with glycolytic, oxidative phosphorylation, and tricarboxylic acid (TCA) cycle were significantly dysregulated between high and low-quality meat, and the color of meat was determined by succinate dehydrogenase (SDH) and nicotinamide adenine dinucleotide dehydrogenase (NADH) [11]. Other studies have shown that the changed expression of proteins associated with the TCA cycle indicated higher oxidative level, poor meat quality, and poor color stability [10, 12, 13].

Beef meat accounts for a high proportion of meat sales worldwide. Among the attributes of beef quality, tenderness is the most important one affecting the acceptability the consumer [14, 15]. Wei et al indicated through proteomics that lactate dehydrogenase (LDH) and heat-shock proteins (HSPs) can be used as markers for tenderness in bovine meat [11]. Others studies demonstrated that proteins such as H2AFX, SUMO4, and TP53 that are involved in metabolism and apoptosis were related with the tenderness of the beef [15–17]. Besides, phosphorylation, glycosylation, and ubiquitination of protein structure affect the activity of many enzymes and subsequently control the tenderness of beef [15, 18]. Our previous study demonstrated that the meat color stability was poor during the storage at 4°C [5]. However, the proteomics-related information on color stability is lacking and the proteomics mechanism during postmortem storage at 4°C is still unclear.

As such, we performed this study to investigate the differential abundance of proteins related to the quality of beef muscle (longissimus dorsi). Muscle samples were stored at 4°C. Proteomics profiling was analyzed and the bioinformatics analysis was performed to identify the biomarkers related to the beef quality.

## Materials and methods

### Sample collection and storage

Beef longissimus dorsi muscles were obtained from a local slaughterhouse and were brought to our laboratory within 4 h after slaughter. The samples were divided into three parts randomly and then stored at 4°C for 0, 12, 24, 36, and 48 h, respectively. They were stored at 100% humidity on sterilized disks without packaging [5].

### Measurement of the beef muscle color, pH and WHC

The changes in color, pH, and WHC during storage were determined according to our previous methods [5]. In brief, a Canon EOS 80D camera with setting parameters was used for imaging of the beef color (Aperture F3.5, Shutter Speed 1/400 sec, ISO 800). WHC in beef muscles was determined using the drip loss as previously reported [5, 19]. WHC was indicated as: Drip loss (%) = 100×(weight of drip/initial weight). Moisture content (%) = 100×(weight loss/initial weight) [5].

### Sarcoplasmic sample preparation

Minced beef was set on sterilized disks at 4°C for 0 d, 3.5 d, and 7 d (3 replications at each time point). After complete grounding, homogenates were then precipitated and incubated in chilled acetone (10% trichloroacetic acid) at -20°C for 2 h. After centrifugation at 4°C and 20,000 g for 20 min (ST16R; Thermo Fisher Scientific, Waltham, MA, USA) the centrifugal precipitates were washed with chilled acetone, lysed in lysis buffer [20], and then subjected to ultrasonic treatment. After double centrifugation (4°C, 20,000 g, and 20 min), the centrifugal supernatant was collected and the protein concentration measured using a BCA Protein Assay Kit (Thermo Scientific, USA). The lysates were used for further analysis immediately or stored at -80°C before use.

### Protein digestion and iTRAQ labeling

Protein aliquots (100 μg) were digested into peptides using 3.3 μg Trypsin (Thermo Scientific.) at 37°C for 24 h, following with centrifugation (at 16000 g, 4°C, 10 min). Peptide precipitation was vacuum-dried, diluted in 0.5 M tetraethylammonium bromide, and then prepared using an iTRAQ kit (Applied Biosystems, Carlsbad, CA, United States) according to the recommendation from the manufacturer. The samples were pooled and vacuum-dried.

### LC-MS/MS analysis

The dried peptide samples were dissolved to form a solution A (10 mM KH_2_PO_4_ in 25% acetonitrile) and trapped onto a strong cation exchange (SCX) column (Luna SCX, 4.6 mm × 250 mm, Phenomenex). The elution of peptides from the trapping column was performed in a gradient buffer B (10 mM KH 2 PO 4 and 1 M KCl in 25% ACN; 1ml/min; 0% for 30 min, 0–5% for 1 min, 5–30% for 20 min, 30–45% for 10 min, and 45–100% for 5 min, and 100% for 20 min). The peptides were trapped onto C18- reversed-phase column (reversed-phase; 200 μm × 2 cm; Thermo Scientific) and eluted onto another reversed-phase C18 column using a gradient buffer B (0.1% formic acid and 98% ACN; 300nl/min; 5%-80% for 45 min). The fractions of the peptides were then collected and subjected to a nanoelectrospray ionization tandem mass spectrometry (MS; Thermo Fisher Scientific; 350–2000 m/z). Data acquisition was performed at 70,000 resolution in full scan mode and 35,000 resolution in MS 2 mode.

### Protein identification

The raw MS/MS data was downloaded and processed using Proteome Discoverer (Thermo Fisher Scientific). Peptides sequences were aligned and searched in the UniProt database (containing 37,609 protein sequences) using the criteria of full tryptic specificity and tolerance of one missed cleavage (15 ppm and 0.02 Da). Proteins with fold discovery rate (FDR) <0.01 were retained and used for the identification of the differentially expressed proteins (DEPs).

### Analysis of DEPs

The DEPs were identified at p-value ≤ 0.05 by the t-test and fold change (FC) ≥ 1.2 or < 0.83. The properties of the DEPs were characterized in Gene Ontology (GO, http://www.geneontology.org), Kyoto Encyclopedia of Genes and Genomes (KEGG, http://www.genome.jp/kegg/) databases and Interpro (IPR) protein domain database at p < 0.05. Heatmap analysis was carried out using heatmap (R package version 0.7.7). Enrichment analysis in GO and KEGG was performed to identify the biological functions and pathways associated with the DEPs, with at p < 0.05.

### Statistical analysis

Statistical analysis was performed in SPSS 22.0 software. Beef quality data were expressed as the mean ± standard deviation. A one-way ANOVA test was performed to analyze the differences among groups, and differences between the two groups were tested using a t-test. p- Value < 0.05 was considered as statistically significant.

## Results

### Profiles of muscle pH, color, and WHC during storage

**Fig 1:**
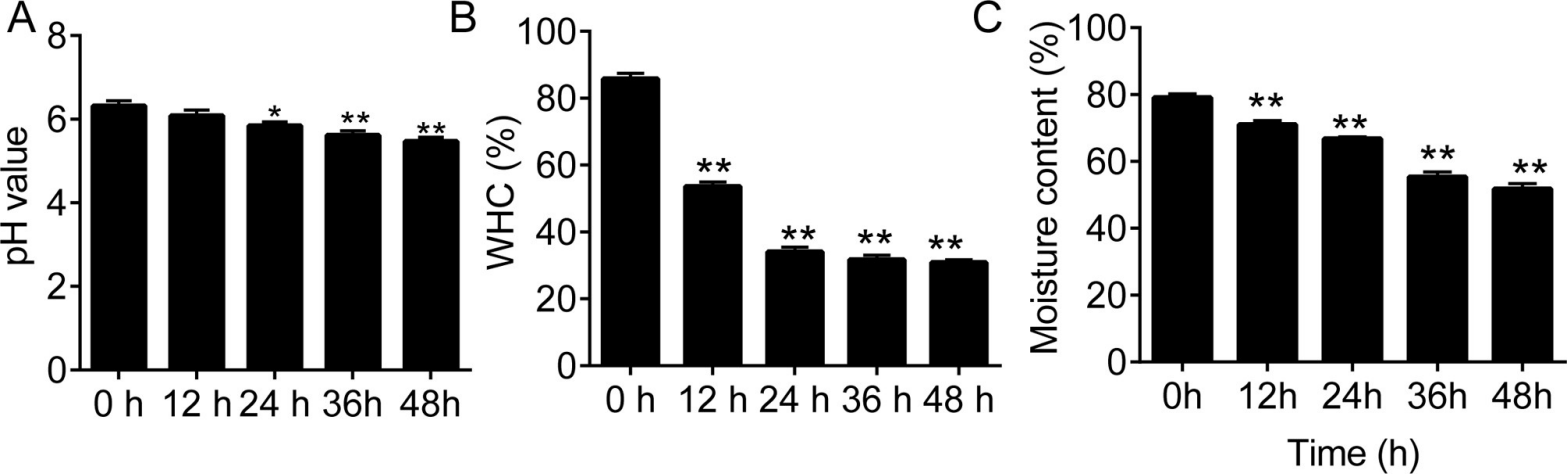
The profiles of beef muscle pH, Water Holding Capacity (WHC), and moisture content during storage. (A), the pH values of the beef muscle samples during storage. (B and C), the water holding capacity (WHC) and moisture content in beef muscle samples during the 48-h storage. RT, 4°C. *p < 0.05 and **p < 0.01 vs. 0 h, respectively. ##, notes p < 0.01 vs. RT at each time point.

Beef muscle pH significantly decreased during the 48 h storage at 4°C (p < 0.01 vs. 0 h; (**Fig 1A**). There was also a significant decrease in WHC and moisture content of beef muscle during the first 24 h storage at 4°C (**Fig 1B**).

### Summary of proteomic data

**Fig 2:**
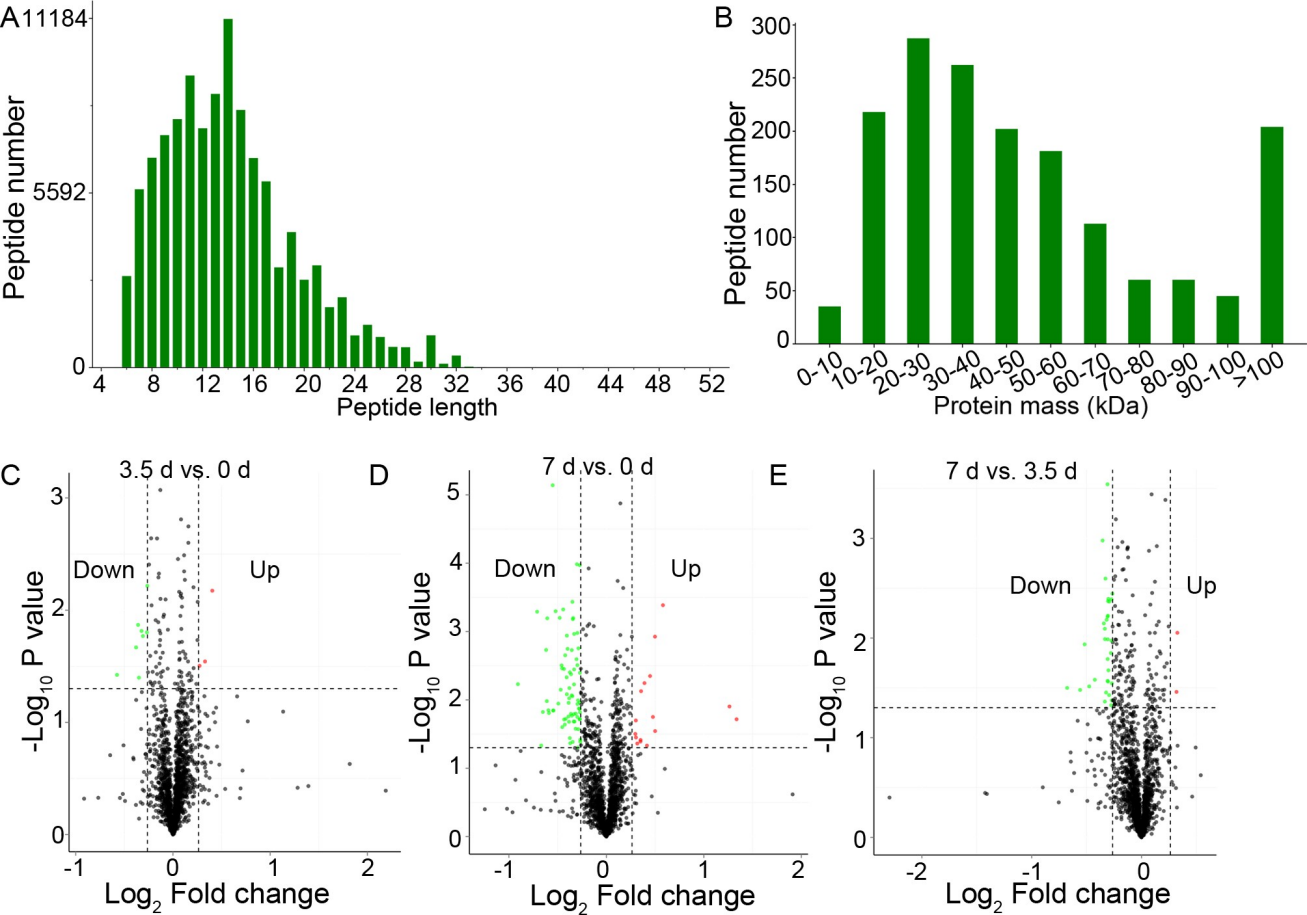
Summary of peptides and proteins, and the Differentially Expressed Proteins (DEPs). (A), the length of the peptides. (B), the mass of the peptides. (C-E), the Volcano plot of the DEPs in beef muscles between 3.5 d and 0 d, 7 d and 0 d, and 7 d and 3.5 d, respectively.

Three samples of 0, 3.5, and 7 d at 4°C storage, respectively, were extracted and used for proteomic analysis. Proteomic data generated 513,662 total spectra, corresponding to 13,345 single peptides and 1,667 proteins. The length of most peptides was between 6 and 32 aa (**Fig 2A**), and the mass of most peptides was between 10 and 100 kDa (**Fig 2B**). Annotation in GO (**S1 Fig**) and KEGG (**S2 Fig**) showed that these proteins were enriched in various biological processes and pathways involved in cellular processes, environmental information processing, and metabolism. The search for InterPro domains resulted in 465 proteins annotated with protein domains including SH3, WD40 repeat, and immunoglobulin (**S3 Fig**).

### Identification of DEPs

**Fig 3:**
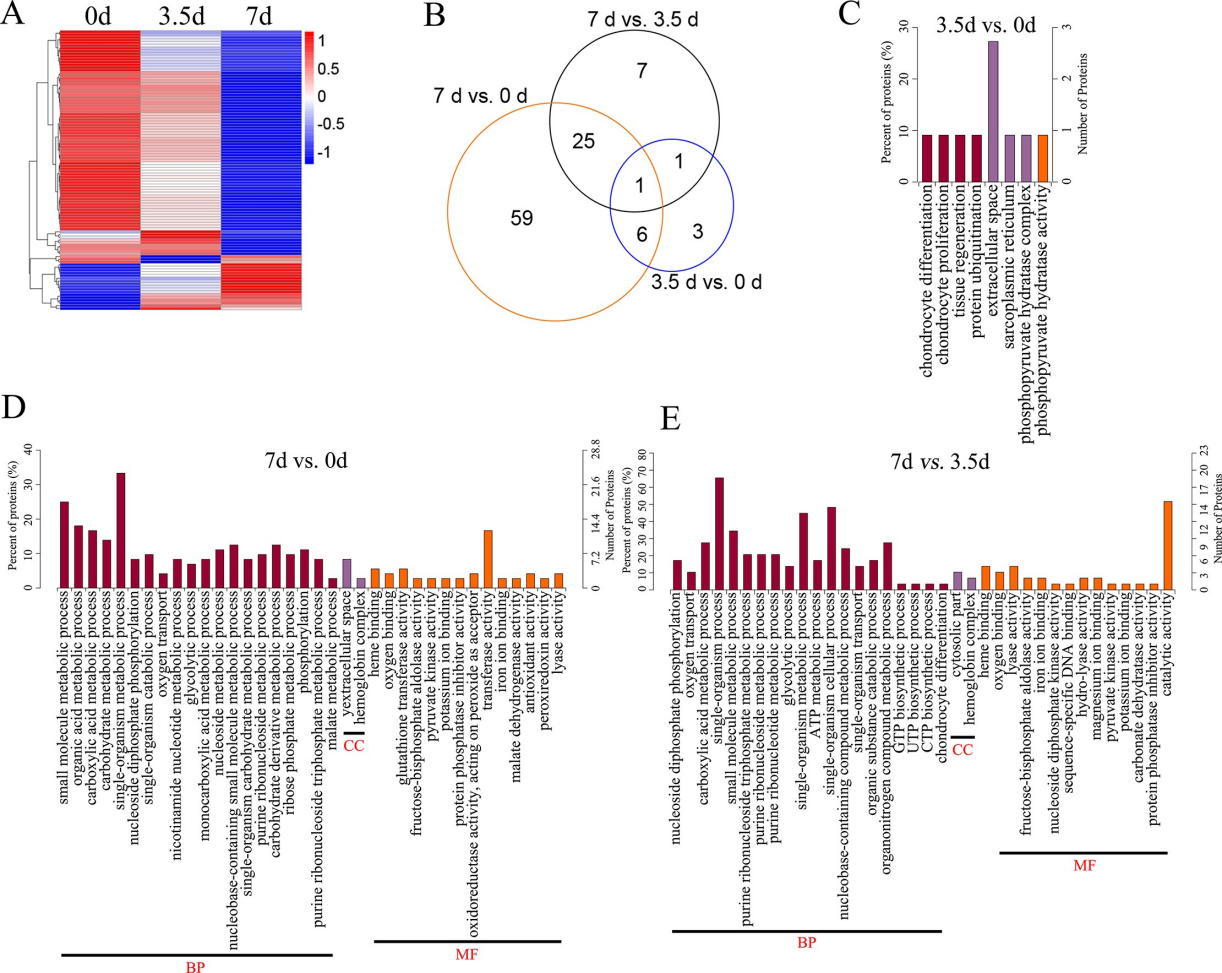
The heatmap, Venn diagram and GO enrichment of the Differentially Expressed Proteins (DEPs). (A), the heatmap illustration of the DEPs. (B), the Venn diagram of the DEPs. (C, D, and E), the enriched GO terms of the DEPs between 3.5 d and 0 d, 7 d and 0 d, and 7 d and 3.5 d, respectively. BP, biological processes. MF, molecular functions. CC, cellular component.

Comparative analysis between groups showed that 102 proteins were differentially expressed between groups, including 11 DEPs between 0 and 3.5 d, 91 DEPs between 0 and 7 d, and 34 DEPs between 3.5 and 7 d, respectively (**Fig 2C–2E and S1 Table**). The bidirectional clustering heatmap showed the significantly differential expression profiles of these DEPs, and most of them gradually decreased during the 7-day storage at 4°C (**Fig 3A**). Venn figure showed that protein Q32KU9 was downregulated at 3.5 d and 7 d compared with 0 d, and at 7 d compared with 3.5 d (**Fig 3B**).

### Properties of the DEPs

DEPs between the samples at 3.5 d and 0 d were associated with chondrocyte differentiation (GO: 0002062; downregulated Q32KU9), chondrocyte proliferation (GO: 0035988; downregulated Q32KU9), sarcoplasmic reticulum (GO: 0016529, upregulated A6QNN1), and phosphopyruvate hydratase complex (GO: 0000015; downregulated A6QR19; **Fig 3C and S2 Table**).

DEPs between samples at 7 d and 0 d, including downregulated A6QLL8, Q3ZC87, F1N2F2, A0A3S5ZPB0, B3IVN4, Q3T145, P33097, and A0A3Q1M5R4, were involved in GO processes like glycolytic process (GO: 0006096), nucleoside metabolic process (GO: 0009116), organic acid metabolic process (GO: 0006082), and organic substance catabolic process (GO: 1901575). A0A3Q1MAU7 was associated with cellular aldehyde metabolic process (GO: 0006081) and small-molecule catabolic process (GO: 0044282; **Fig 3D and S2 Table**).

DEPs between samples at 7 d and 3.5 d were enriched into the carboxylic acid metabolic process (GO: 0019752), and organic substance catabolic process (GO: 1901575; including A6QLL8, Q3ZC87, A0A3S5ZPB0, Q3T145, P33097 and A0A3Q1M5R4), UTP biosynthetic process (GO: 0006228; downregulated Q3T0Q4), glycolytic process (GO: 0006096; upregulated A6QR19 and downregulated A6QLL8, Q3ZC87, and A0A3S5ZPB0), oxygen transport (GO: 0015671; downregulated A0A1K0FUF3, D4QBB4, and P01966, **Fig 3E and S2 Table**).

### KEGG pathways associated with DEPs

**Fig 4:**
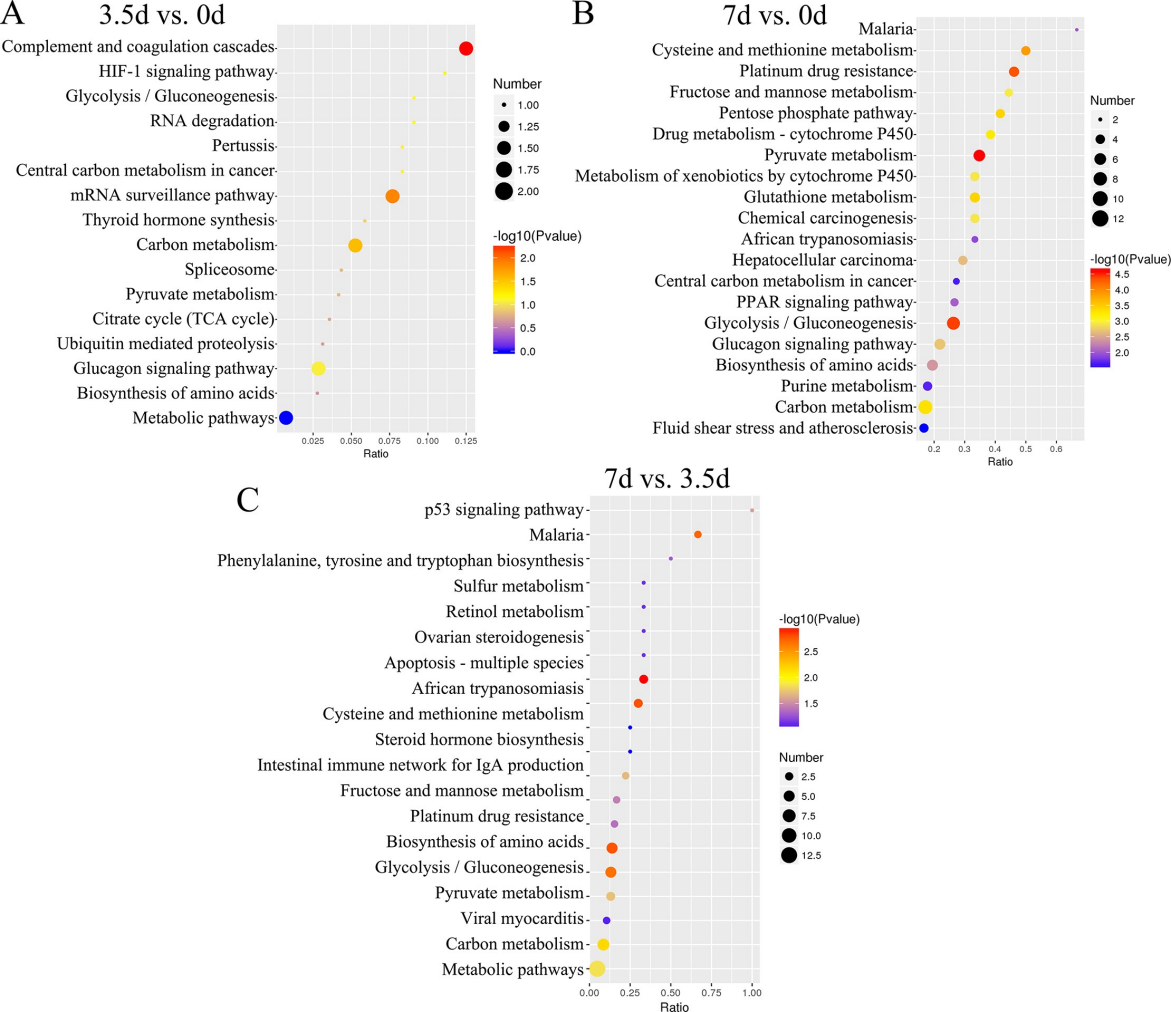
The KEGG enrichment pathway of the Differentially Expressed Proteins (DEPs). (A, B, and C), the KEGG pathways associated with DEPs between 3.5 d and 0 d, 7 d and 0 d, and 7 d and 3.5 d, respectively.

KEGG pathways enrichment revealed that DEPs between the samples at 3.5 d and 0 d were associated with three pathways HIF-1 signaling pathway (map04066; downregulated A6QR19 and Q2T9Y3; **Fig 4A and S3 Table**), Glycolysis/Gluconeogenesis (map00010; A6QR19 and Q2T9Y3), and Complement and coagulation cascades (map04610; downregulated P34955 and E1BMJ0). DEPs between samples at 7 d and 0 d, and 7 d and 3.5 d were associated with Malaria (map05144; downregulated D4QBB4 and P01966), Pyruvate metabolism (map00620; downregulated Q3ZC87, Q3T145, and A0A3Q1M5R4), Glucagon signaling pathway (map04922) and Biosynthesis of amino acids (map01230; A6QLL8, Q3ZC87, F1N2F2, A0A3S5ZPB0, P33097, B3IVN4, and G5E5C8; **Fig 4B and 4C, and S3 Table**). DEPs between 7 d and 0 d were also associated with the PPAR signaling pathway (map03320; including downregulated P10790, P55052, and Q58DK1; **Fig 4C, and S3 Table**).

### Key DEPs in the PPI network

**Fig 5:**
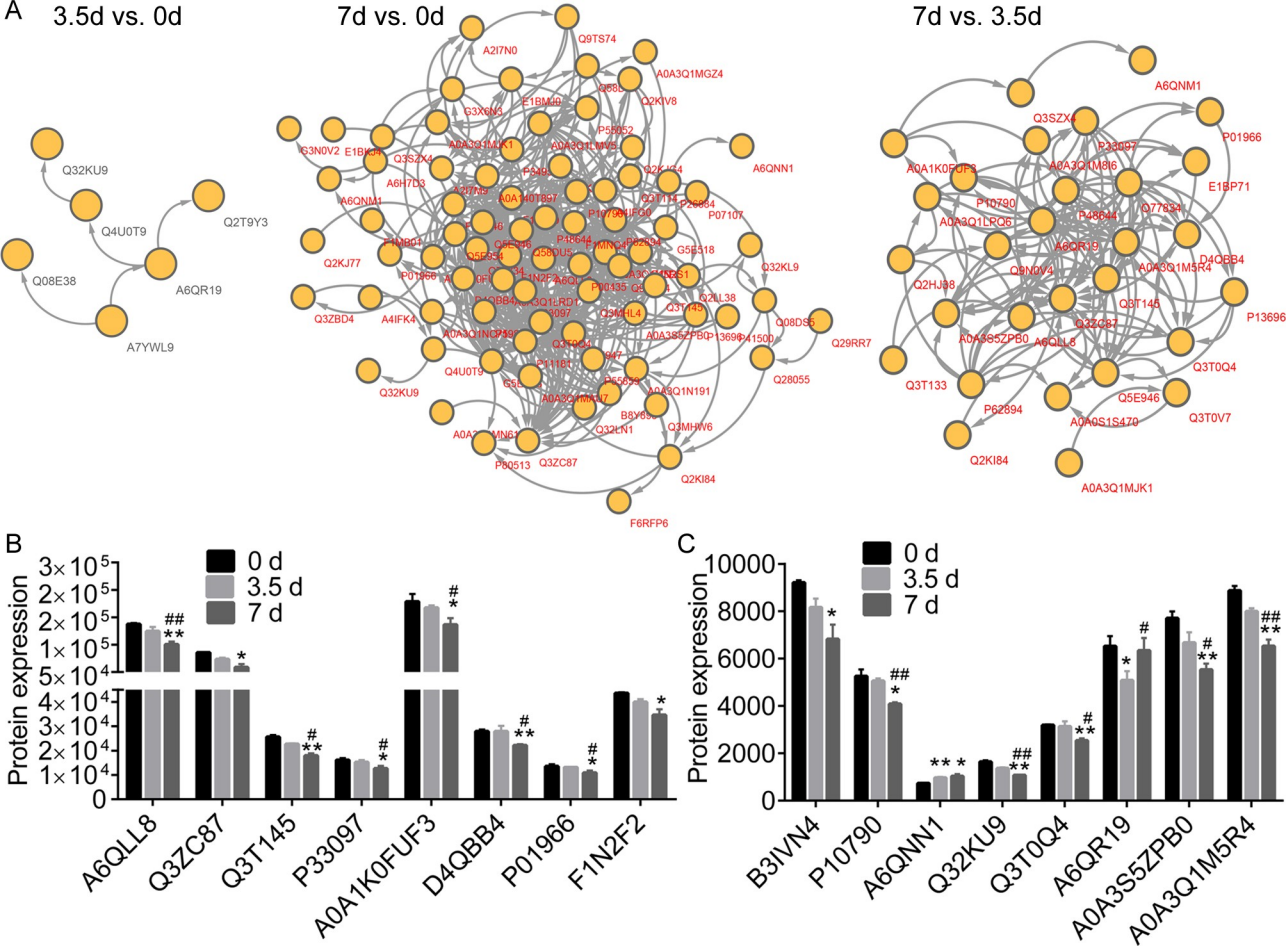
The Protein-Protein Interaction (PPI) network and expression profiles. (A), the PPI network of the differentially expressed proteins (DEPs) between different time points. (B and C), the expression profiles of several DEPs in meat samples post storage at 4°C. * and ** notes p < 0.05 and p < 0.01 vs. 0 d, respectively. # and ## indicates p < 0.05 and p < 0.01 vs. 3.5 d, respectively.

PPI network identified key DEPs between meat samples stored for 0 d, 3.5 d, and 7 d (**Fig 5A**). We realized that the downregulated Q32KU9 and A6QR19, and upregulated Q4U0T9 were key proteins between samples stored for 3.5 d and 0 d. A6QLL8, Q3ZC87, A0A3Q1M5R4, A6QR19, A6QNN1, A0A1K0FUF3, Q3T145, Q3T0Q4, P01966, and P33097 were key proteins in the PPI consisting of the DEPs between 7 d and 0 d. A6QLL8, Q3ZC87, A0A3Q1M5R4, A6QR19, A0A1K0FUF3, Q3T145, Q3T0Q4, P01966, and P33097 were associated with the PPI consisting of DEPs between 7 d and 3.50 d.

### Description of the key DEPs

Table 1 shows the descriptions for the DEPs that were the GO as mentioned above terms and KEGG pathways associated with organic acid and energy metabolic processes. These include glycolysis/gluconeogenesis (map00010), glycolytic process (GO: 0006096), and organic acid metabolic process (GO: 0006082). The expression of A6QLL8 (Fructose-bisphosphate aldolase), A0A3S5ZPB0 (Aldolase, fructose-bisphosphate C), A0A1K0FUF3 (Myoglobin), A0A3Q1M5R4 (L-lactate Dehydrogenase B chain, LDH), P01966 (Hemoglobin subunit alpha), P10790 (Fatty acid-binding protein, heart), P33097 (Aspartate aminotransferase), Q32KU9 (Musculoskeletal embryonic nuclear protein 1), and Q3T145 (Malate dehydrogenase, cytoplasmic, MDH1) were consistently downregulated in muscle samples collected at 3.5 d and 7 d. The expression profiles of various DEPs were as shown in **Fig 5B and 5C**.

**Table 1:** Description of several key differentially expressed proteins.

Protein	Description	Gene	3.5 d vs. 0 d	7 d vs. 0 d	7 d vs. 3.5 d	Function
A6QR19	ENO2 protein	ENO2	Down	NA	Up	phosphopyruvate hydratase complex; glycolytic process; enolase
A6QNN1	TRDN protein	TRDN	Up	Up	NA	sarcoplasmic reticulum; receptor binding
Q4U0T9	Cysteine and glycine-rich protein 3	CSRP3	Up	Up	NA	zinc ion binding
A6QLL8	Fructose-bisphosphate aldolase	ALDOA	NA	Down	Down	glycolytic process
A0A3S5ZPB0	Aldolase, fructose-bisphosphate C	ALDOC	NA	Down	Down	Pentose phosphate pathway; Fructose and mannose metabolism; Biosynthesis of amino acids
A0A1K0FUF3	Myoglobin	GLNG	NA	Down	Down	oxygen transport; oxygen binding;
A0A3Q1M5R4	L-lactate dehydrogenase B chain	LDHB	NA	Down	Down	oxidoreductase activity; oxidation-reduction process
B3IVN4	M1-type pyruvate kinase (Fragment)	PKM	NA	Down	NA	magnesium ion binding; pyruvate kinase activity; glycolytic process;
D4QBB4	Globin A1	HBB	NA	Down	Down	iron ion binding; hemoglobin complex; oxygen transport; heme binding
E1BMJ0	Uncharacterized protein	SERPING1	Down	Down	NA	extracellular space; C1 inhibitor; Complement and coagulation cascades
F1N2F2	Phosphoglycerate mutase	PGAM2	NA	Down	NA	phosphoglycerate mutase activity; glycolytic process;
G5E5C8	Transaldolase	TALDO1	NA	Down	NA	pentose-phosphate shunt
P01966	Hemoglobin subunit alpha	HBA	NA	Down	Down	hemoglobin complex; oxygen transport; oxygen binding; heme binding
P10790	Fatty acid-binding protein, heart	FABP3	NA	Down	Down	transporter activity; lipid binding
P55052	Fatty acid-binding protein 5	FABP5	NA	Down	NA	transporter activity; lipid binding
P34955	Alpha-1-antiproteinase	SERPINA1	Down	Down	NA	extracellular space; Complement and coagulation cascades
P33097	Aspartate aminotransferase	GOT1	NA	Down	Down	cellular amino acid metabolic process; transaminase activity
Q2T9Y3	Pyruvate dehydrogenase E1 component subunit alpha	PDHA2	Down	NA	NA	metabolic process
Q32KU9	Musculoskeletal embryonic nuclear protein 1	MUSTN1	Down	Down	Down	chondrocyte differentiation/proliferation; tissue regeneration
Q3ZC87	Pyruvate kinase (Fragment)	PKM2	NA	Down	Down	magnesium ion binding; pyruvate kinase activity; glycolytic process
Q3T0Q4	Nucleoside diphosphate kinase B	NME2	NA	Down	Down	nucleoside diphosphate kinase activity; GTP biosynthetic process
Q3T145	Malate dehydrogenase, cytoplasmic	MDH1	NA	Down	Down	malate metabolic process; oxidoreductase activity
Q58DK1	Carnitine O-palmitoyltransferase 1, muscle isoform	CPT1B	NA	Down	NA	Fatty acid degradation

NA, not applicable.

## Discussion

Many proteomic studies have been performed and brought about new insights on postmortem storage. The studies identified protein biomarkers associated with the tenderness, color stability, and flavor of meat [8, 10, 15, 17, 21, 22]. Our current study showed that the temperature; an extrinsic factor, influenced the beef quality. The pH value, WHC, and moisture content in beef muscle were gradually decreased during storage at 4°C. Proteome analysis indicated that a cluster of proteins, including proteins involved in glycolysis/gluconeogenesis, oxygen transport, and phosphopyruvate hydratase complex, was gradually decreased during the 4°C storage.

Herein, we found downregulated expression of proteins related to glycolytic enzymes, including ENO2, LDH, PGM, and PKM. PKM catalyzes the irreversible process in glycolysis involving ATP generation from phosphoenolpyruvate [23–25]. Malate dehydrogenase (MDH1) is a key enzyme of the TCA cycle and is involved in NADH production which is crucial for energy metabolism. ENO2 (Enolase) has been reported to promote cellular glycolysis in lymphoblastic leukemia [26]. Other studies revealed that ENO1and3 are protein markers for tenderness in goat meat [11, 15]. Wei et al reported that the downregulated proteins including LDH, NADH, and phosphatase-3-phosphate dehydrogenase (PGM) in low-quality goat meat were associated with glycolysis and the TCA cycle. The positive association of these proteins with the tenderness of beef have been confirmed in previous studies [27–29]. Accordingly, we realized that the decreased expression of glycolytic enzymes including LDH, ENO2, MDH and PKM was associated with the decreased tenderness of minced beef.

During the postmortem storage of the meat, the glycolytic process was gradually decreased, whereas the lactate concentration was increased [30]. The downregulation of these proteins (LDH, ENO2, MDH, and PKM) during meat storage indicated reduced glycolysis. Decreased glycolysis directly or indirectly facilitates the production and accumulation of organic acids and the associated intermediate metabolites of the TCA cycle, including lactate, malate, and pyruvate. The metabolites are endogenous to the skeletal muscles [31]. Malate accumulation minimizes the formation of metmyoglobin in the beef muscle [32]. Lactate is a substrate for mitochondrial respiration. It increases oxygen consumption in muscle cells, and hence induce beef color darkening under unlimited oxygen conditions [32]. The accumulation of lactate, malate, and pyruvate in beef had been reported to reduce metmyoglobin and increase the stability of myoglobin redox form [33]. The increased acidity in beef on the other hand impact its color stability.

Another factor that influenced meat color and color stability was hemoglobin. Hemoglobin is the main iron-binding protein and oxygen transporter in the serum, while myoglobin facilitates oxygen diffusion into muscle cells [34]. Hemoglobin is a major pigment in beef [35, 36]. Hemoglobin is attributed to the meat color and is abundant in meat with dark color [36, 37]. The abundance of hemoglobin is correlated with higher blood flow and oxidative status in the muscles [38]. The oxidation of hemoglobin and myoglobin contribute to meat browning [39–42]. Besides, the cluster of proteins associated with the redox process, and glycolytic metabolism are correlated with the beef color stability during postmortem aging [1, 9, 12]. Wu et al observed that the glycolytic enzymes including glycerol-3-phosphate dehydrogenase, glycogen-phosphorylase and phosphoglucomutase-1 could predict meat color stability during postmortem aging [21]. Our current study revealed the decreased expression of hemoglobin complex members (Hemoglobin subunit alpha, P01966; and Globin A1, D4QBB4) and myoglobin (A0A1K0FUF3) at 3.5 d and 7 d during beef meat postmortem storage. These findings suggested that the increased acidity and decreased glycolytic enzymes were in line with the meat color stability.

## Conclusion

In summary, the considerable impact of 4°C storage on beef quality and proteomics was confirmed. The decreased expression of glycolytic enzymes including MDH1, PKM, and PGM, and the heme pigments including hemoglobin and myoglobin were correlated with decreased pH value and WHC. These proteins might be used as biomarkers for the decreased tenderness and poor color stability. This study, therefore, provides novel insights into the proteomics of beef quality.

## Supporting information

S1 Fig The top 20 terms of GO enrichment of the total proteins. (TIF)

S2 Fig The KEGG pathways associated with the total proteins. (TIF)

S3 Fig Protein domain properties annotated InterPro database. (TIF)

S1 Table The list of the differentially expression proteins between different time points at 4°C storage. (XLSX)

S2 Table GO terms associated with the differentially expressed proteins between different time point at 4°C storage. (XLSX)

S3 Table KEGG pathways associated with the differentially expressed proteins between different time point at 4°C storage. (XLSX)

S1 File (PDF)
