# Supplementary figures and images for: Application of proteomics to understand the molecular mechanisms determining meat quality of beef muscles during postmortem aging
Source: PLoS One. 2021 Mar 1;16(3):e0246955. doi: 10.1371/journal.pone.0246955 (PMC7920376; doi:10.1371/journal.pone.0246955)

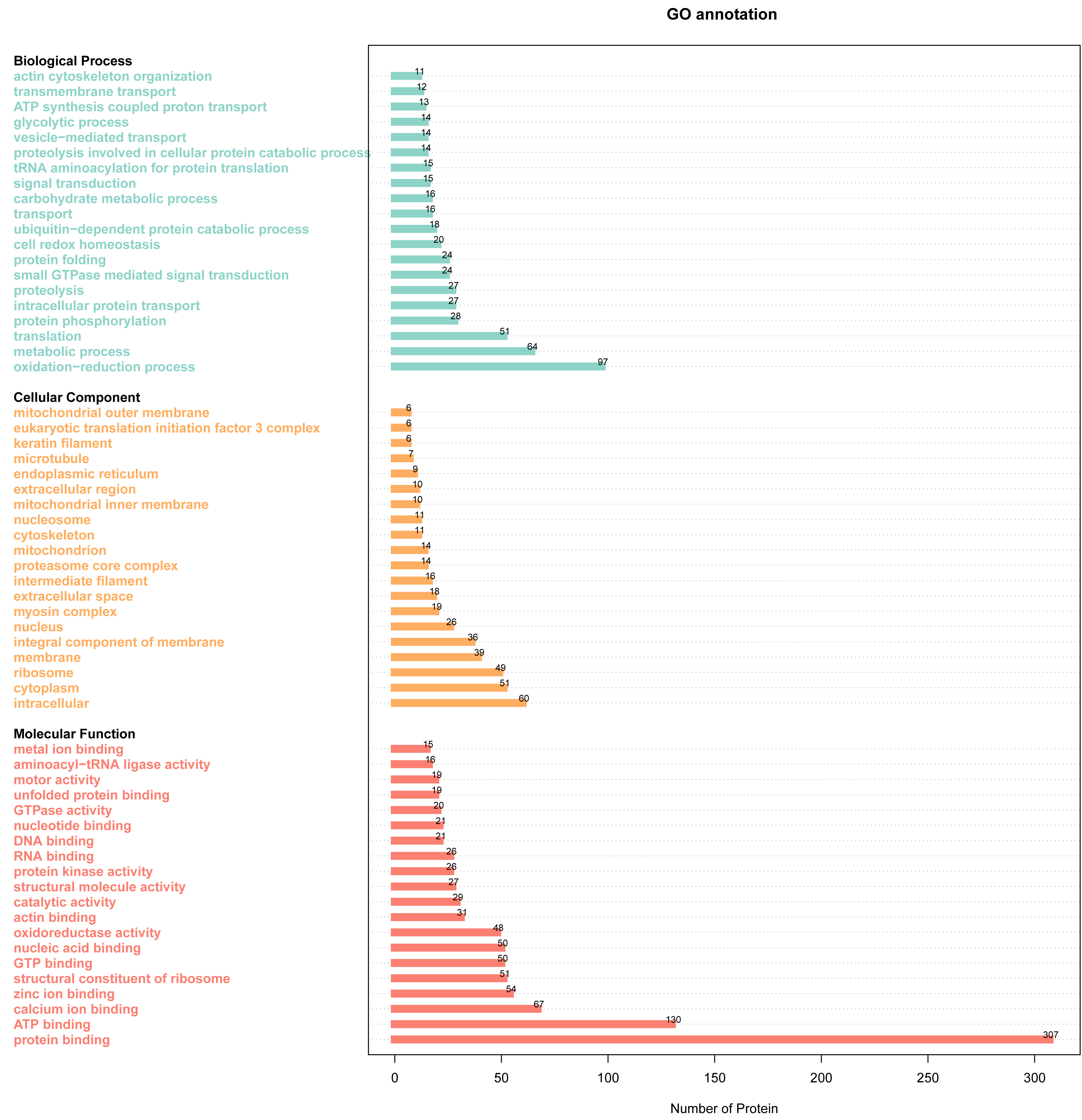

Supplement: S1 Fig — (TIF) [file pone.0246955.s001.tif]

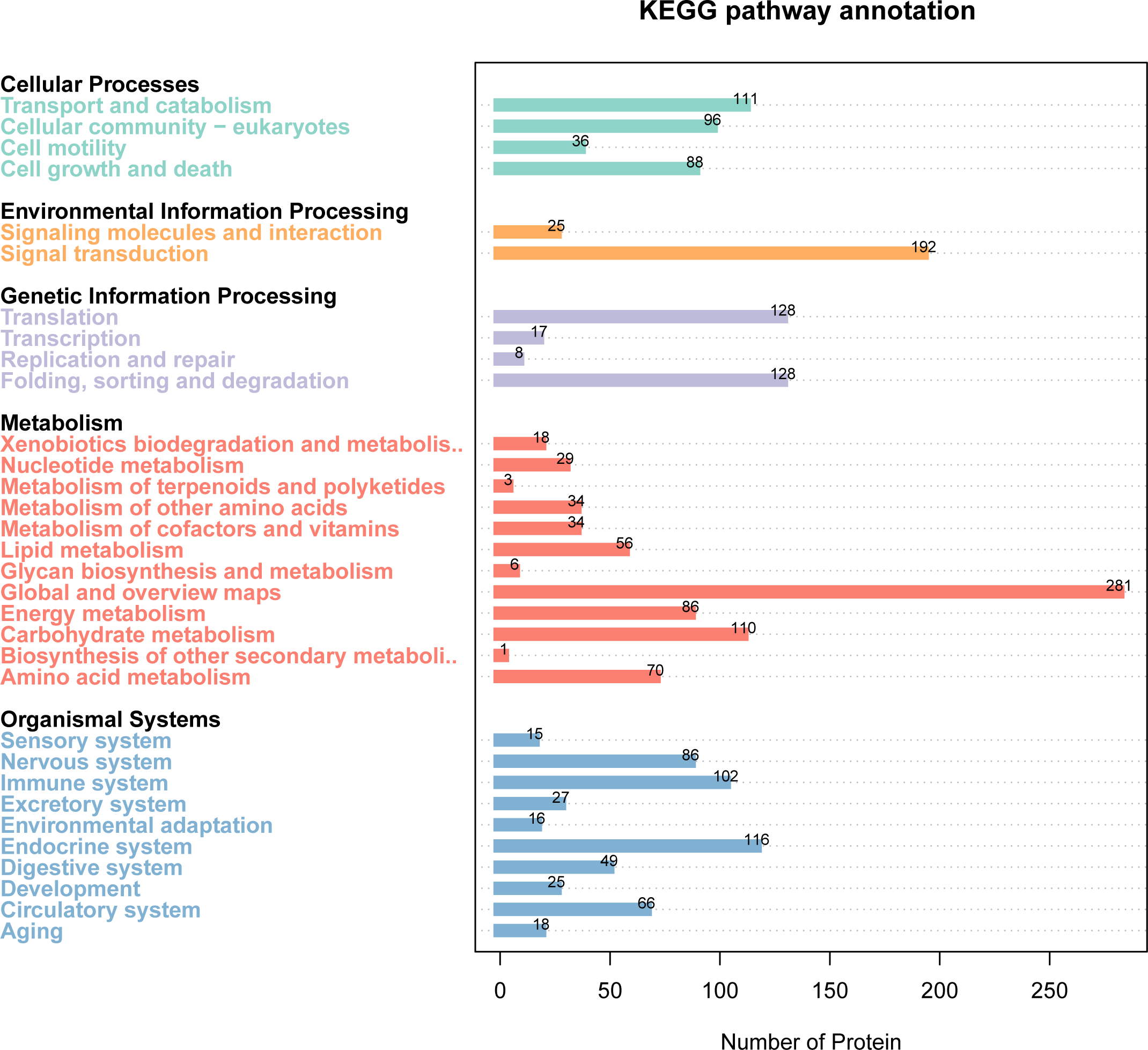

Supplement: S2 Fig — (TIF) [file pone.0246955.s002.tif]

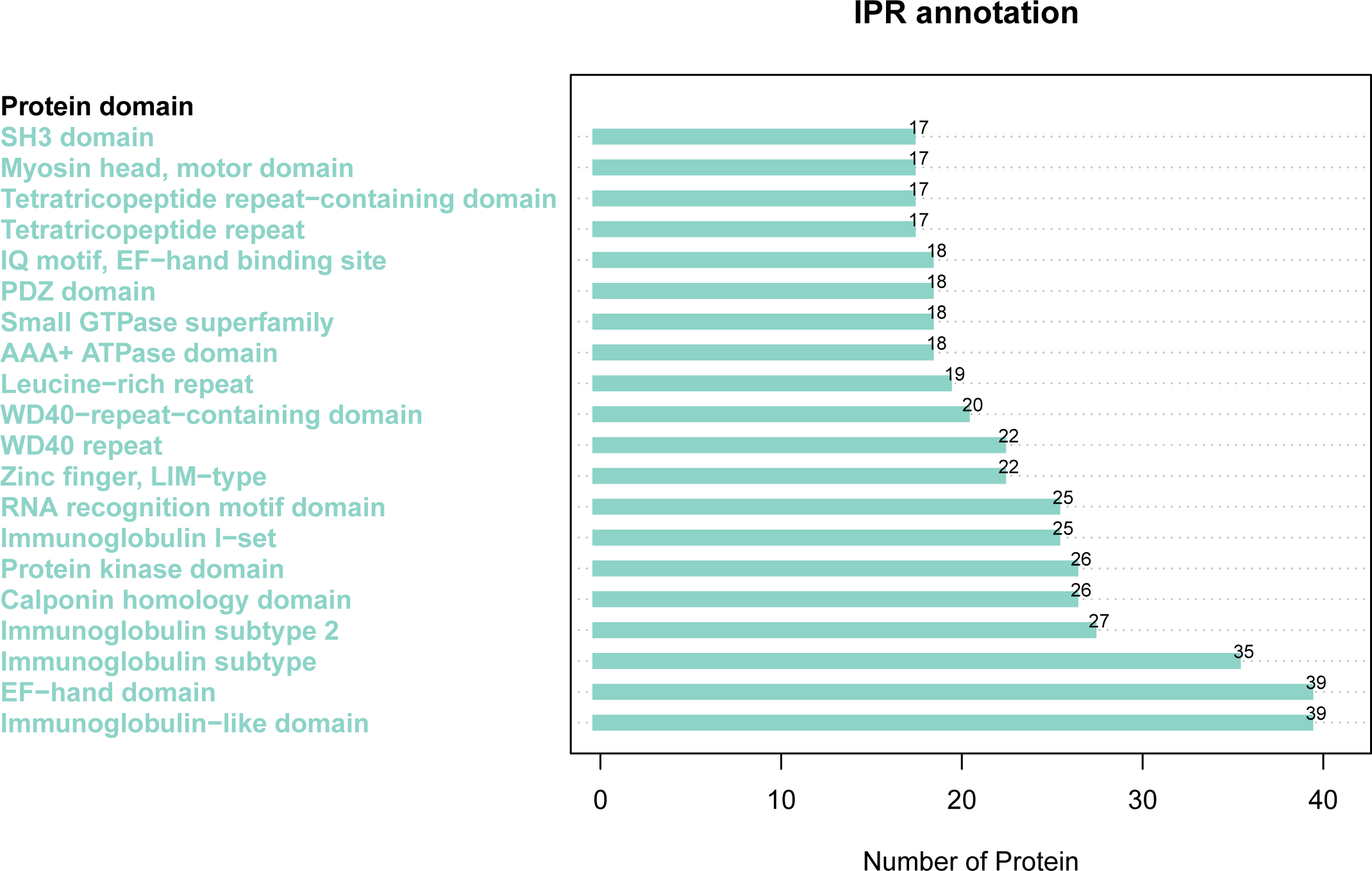

Supplement: S3 Fig — (TIF) [file pone.0246955.s003.tif]
