# Supplementary material for: Application of proteomics to understand the molecular mechanisms determining meat quality of beef muscles during postmortem aging
Source: PLoS One. 2021 Mar 1;16(3):e0246955. doi: 10.1371/journal.pone.0246955 (PMC7920376; doi:10.1371/journal.pone.0246955)
Supplement: S1 File — (PDF) [file pone.0246955.s007.pdf]

To whom it may concern,

This letter is to certify that the manuscript titled "**Application of proteomics to understand the molecular mechanisms determining meat quality of beef muscles during postmortem aging**" has been proof read and corrected by the Home for researchers editorial team on the 09/05/2020. After being checked and amended as seen appropriate, we feel that the standard of English in this manuscript satisfies the requirements of submission to journals to be considered for publication.

Sincerely,
Home for researchers editorial team

Guosheng Wang
